# Salivary microbiota and inflammation‐related proteins in patients with psoriasis

**DOI:** 10.1111/odi.13277

**Published:** 2020-01-28

**Authors:** Daniel Belstrøm, Josefine Maria Eiberg, Christian Enevold, Maria Anastasia Grande, Claus Antonio Juel Jensen, Lone Skov, Peter Riis Hansen

**Affiliations:** ^1^ Section for Periodontology and Microbiology Department of Odontology Faculty of Health and Medical Sciences University of Copenhagen Copenhagen Denmark; ^2^ Institute for Inflammation Research Center for Rheumatology and Spine Diseases Rigshospitalet Copenhagen University Hospital Copenhagen Denmark; ^3^ Department of Clinical Biochemistry Nordsjællands Hospital Hillerød Denmark; ^4^ Department of Dermatology and Allergy Faculty of Health and Medical Sciences Herlev and Gentofte Hospital University of Copenhagen Hellerup Denmark; ^5^ Department of Cardiology Herlev and Gentofte Hospital Hellerup Denmark

**Keywords:** inflammation, microbiota, periodontitis, psoriasis, saliva

## Abstract

**Objective:**

The purpose of the present study was to characterize the composition of the salivary microbiota and quantify salivary levels of inflammation‐related proteins (neutrophil gelatinase‐associated lipocalin [NGAL] and transferrin) in patients with psoriasis and compare data to those obtained in patients with periodontitis and orally healthy controls, respectively.

**Materials and methods:**

Stimulated saliva samples from patients with psoriasis (*n* = 27), patients with periodontitis (*n* = 58), and orally healthy controls (*n* = 52) were characterized by means of next‐generation sequencing of the 16S rRNA gene. Salivary levels of NGAL and transferrin were quantified using immunoassays.

**Results:**

Linear discriminant effect size analysis showed that 52 (22 psoriasis‐associated and 30 periodontitis‐associated) and 21 (8 psoriasis‐associated and 13 orally healthy control‐associated) bacterial taxa differentiated the salivary microbiota in patients with psoriasis from that of patients with periodontitis and orally healthy controls, respectively. Significantly lower mean salivary levels of NGAL (psoriasis: 996 [std. error 320], periodontitis: 2,072 [295], orally healthy controls: 2,551 [345] ng/ml, *p* < .0001) and transferrin (psoriasis: 4.37 [0.92], periodontitis: 7.25 [0.88], orally healthy controls: 10.02 [0.94] ng/ml, *p* < .0001) were identified in patients with psoriasis.

**Conclusions:**

Psoriasis associates with characteristics of the salivary microbiota and salivary levels of inflammation‐related proteins, which are different from characteristics in patients with periodontitis and orally healthy controls, respectively.

## 1 INTRODUCTION

Psoriasis is a chronic inflammatory disease, with a prevalence of approximately 2% in the western world (Nestle, Kaplan, & Barker, 2009). The pathobiological mechanisms underlying psoriasis are not fully understood, but the driving factor is presumably aberrant activation of both the innate and the adaptive immune system, which causes characteristic local inflammatory reactions in the skin as well as systemic low‐grade inflammation with increased circulating levels of inflammatory cytokines (Dowlatshahi, Voort, Arends, & Nijsten, 2013; Mahil, Capon, & Barker, 2015; Nestle et al., 2009). Periodontitis is a chronic inflammatory disease of the tooth supporting tissues, which affects as much as 50% of the adult population in the western world (Eke, Dye, Wei, Thornton‐Evans, & Genco, 2012). The hallmark of periodontitis is a chronic inflammatory reaction in the periodontal tissue that is driven, in part, by responses directed at the oral microbiota (Bartold & Van Dyke, 2017).

Several cross‐sectional studies have shown a higher prevalence of periodontitis and increased periodontal disease activity in patients with psoriasis, as compared to orally healthy controls (Egeberg, Mallbris, Gislason, Hansen, & Mrowietz, 2017; Lazaridou et al., 2013; Preus, Khanifam, Kolltveit, Mørk, & Gjermo, 2010; Sharma, Raman, & Pradeep, 2015; Skudutyte‐Rysstad, Slevolden, Hansen, Sandvik, & Preus, 2014; Üstün et al., 2013; Woeste, Graetz, Gerdes, & Mrowietz, 2019). Recently, these findings were confirmed in a systematic review and meta‐analysis, which reported a significant psoriasis‐associated risk of periodontitis (Ungprasert, Wijarnpreecha, & Wetter, 2017). Conversely, patients with psoriasis appear to have an increased risk of periodontitis (Keller & Lin, 2012; Nakib, Han, Li, Joshipura, & Qureshi, 2013). Collectively, these studies indicate a bi‐directional relationship between periodontitis and psoriasis, with one disease increasing risk of the other, and vice versa. Although shared inflammatory mechanisms may contribute to this relationship, the biological mechanisms behind these findings remain unclear.

Saliva is the fluid of the oral cavity, which besides being critical to maintenance of oral homeostasis harbors various biological substances such as the salivary microbiota and inflammatory markers (Lynge Pedersen & Belstrom, 2019). The presence of untreated periodontitis has been shown to alter the composition of the salivary microbiota (Belstrom et al., 2017) and increase salivary levels of inflammatory proteins such interleukin (IL)‐1β and matrix metalloproteinase (MMP)‐8 (Lee, Chen, Tu, Wu, & Chang, 2018; Liukkonen, Gürsoy, Pussinen, Suominen, & Könönen, 2016; Sorsa et al., 2016). Thus, the composition of the salivary microbiota and salivary levels of inflammatory markers have been suggested as a proxy of oral and systemic health status (Yoshizawa et al., 2013). While psoriasis associates with periodontitis, it is not known whether psoriasis directly impacts these salivary markers of oral homeostasis.

The purpose of the present study was therefore to characterize the composition of the salivary microbiota in patients with psoriasis. Furthermore, as inflammatory mechanisms including neutrophils and presumably iron metabolism play important roles in psoriasis (Rocha‐Pereira et al., 2004; Schon, Broekaert, & Erpenbeck, 2017) we aimed to quantify salivary levels of the inflammation‐related proteins, neutrophil gelatinase‐associated lipocalin (NGAL) and transferrin in patients with psoriasis. We compared these data to those in patients with periodontitis and orally healthy controls, respectively, to test the hypothesis that psoriasis is linked with characteristics of the salivary microbiota and salivary levels of inflammation‐related proteins, which are different from characteristics in patients with periodontitis and oral healthy individuals, respectively.

## 2 METHODS

### 2.1 Study population

In the period from October 2018 to May 2019, a total of 142 individuals were screened for enrollment in the study. Patients with psoriasis were recruited at the Department of Dermatology, Herlev and Gentofte Hospital, whereas patients with periodontitis and orally healthy controls were recruited at the Department of Odontology, University of Copenhagen. General inclusion criteria were age > 18 years and ≥20 remaining natural teeth. General exclusion criteria included pregnancy, breast‐feeding, and use of any systemic antibiotics 3 months prior to study participation. Patients with psoriasis were excluded if they had treatment‐requiring periodontitis, and patients with periodontitis were excluded if they had psoriasis. Accordingly, 13 patients with psoriasis were excluded as they had treatment‐requiring periodontitis, and samples from 7 patients with psoriasis were excluded as they did not pass initial salivary sample quality control due to insufficient amount of bacterial DNA. Consequently, samples from 27 patients with psoriasis, 58 patients with periodontitis, and 52 orally healthy controls were included in the study. The study was performed in accordance with the Helsinki declaration, and all participants signed an informed consent prior to participation. The study was approved by the regional ethical committee (H‐18013543) and reported to the local data authorization of the Faculty of Health and Medical Sciences, University of Copenhagen.

### 2.2 Clinical examination and case definitions

Periodontal examination was performed in all patients with psoriasis by the same examiner (JME), and plaque, bleeding on probing (BOP), probing pocket depth (PPD), and clinical attachment level (CAL) were recorded at six sites per tooth (third molars excluded). Periodontitis was defined as BOP ≥ 25% of total sites, with minimum two teeth with clinical attachment level ≥ 4 mm and a minimum two teeth with PPD ≥ 6 mm (Kongstad et al., 2013), corresponding to stage 3 using the newly presented classification of periodontitis (Papapanou et al., 2018).

### 2.3 Collection of saliva samples

Stimulated saliva samples were collected between 8.00 a.m. and 15.00 p.m. Monday–Friday. The saliva samples were collected at least two hours after self‐performed oral hygiene, and before any dental treatment to avoid bleeding and contamination. Two types of stimulated saliva samples were collected. First, a Salivette (Sarstedt) was used. The swab from the Salivette was placed in the mouth of each of the participants. The swab was removed after 2 min and stored in the suspended insert of the Salivette. Two Salivette saliva samples were collected from each participant. Next, a paraffin‐stimulated saliva sample was collected as previously described (Bardow et al., 2014). The saliva was collected in plastic cups and split into two aliquots, which were transferred to Eppendorf tubes (Eppendorf Nordic). The Salivettes and the Eppendorf tubes were stored at –80° Celsius degrees until analysis.

### 2.4 Sequencing and data analysis

Bacterial 16S rRNA gene‐targeted amplicon sequencing was performed using a custom dual‐index protocol, as previously described (Kozich, Westcott, Baxter, Highlander, & Schloss, 2013). Custom 16S primers were used to amplify the V1‐V3 regions of the 16S rRNA gene, which were designed to provide the best coverage of the 16S gene while maintaining high sensitivity. Libraries were prepared using a 22‐cycle PCR, which reduces chimera formation (unless otherwise noted). Purification of final PCR products was done using Ampure XP beads, pooled in equal amounts, and gels were purified by the QIAGEN MinElute Gel Extraction Kit (Qiagen). Quantification of pooled purified libraries was performed using the NEBNext Library Quant Kit for Illumina (Illumina). The Illumina^®^ MiSeq was used for sequencing of final libraries with a v2 reagent kit (500 cycles) at a 10 p.m. loading concentration with >20% PhiX spike‐in. The DADA2 R package (Callahan et al., 2016) was used to identify and quantify amplicon sequencing reads on the FastQC files obtained after demultiplexing with the Illumina MiSeq software, and low‐quality sequences were removed using FastQC. Results of FastQC were compiled using MultiQC (Ewels, Magnusson, Lundin, & Käller, 2016). Trimmed and filtered reads were processed through the denoizing, concatenating read1 and read2 with a 10N spacer, and chimera removal steps of DADA2 to identify and quantify true amplicon sequence variants (ASV) present in the sample. Taxonomy of the identified ASVs was assigned using the RDP classifier algorithm (Wang, Garrity, Tiedje, & Cole, 2007) implemented in the DADA2 package with a training dataset developed at The Forsyth Institute and based on the Extended Human Oral Microbe Database (eHOMD) (Chen et al., 2010).

### 2.5 Analyses of NGAL and transferrin

The samples were thawed at RT, spun down at 2000G for 5 min, and transferred to standard 13‐mm analysis tubes. NGAL was analyzed on the Siemens Vista 1500 platform (Siemens) using a NGAL Test assay (cat. nr. ST001RA), the NGAL Calibrator Kit (cat. nr. ST002CA), and the NGAL Control Kit (cat. nr ST003CA) all from Bioporto (Bioporto Diagnostics). Samples with NGAL concentration over 3,000 ng/ml were diluted 1:4, 1:9, or 1:19 in isotonic NaCl and reanalyzed. Transferrin concentration in saliva was measured on the Vista 1500 platform using the TRF Flex reagent in urine mode, as the transferrin concentration in saliva is low. The detection limit of NGAL and transferrin was 25 ng/ml and 2.46 mg/L, respectively. In case of measurements below detection limit, the value was set conservatively as the detection limit.

### 2.6 Statistics

Since no previous data were available on salivary levels of NGAL and transferrin or the salivary microbiota in patients with psoriasis, the sample size was calculated using the clinical periodontal parameter, mean PPD. Based on findings from previous studies (Belstrøm et al., 2018; Üstün et al., 2013), the expected PPD in the periodontitis group was 3.4 ± 1.0 mm and the expected PPD in the psoriasis group was 2.8 mm. With fixed values of *α* = 0.05 and *β* = 0.10, the calculated sample size was *n* = 116, with 58 individuals in each group. Thus, the number of healthy controls was also set to *n* = 58. All parameters tested were checked for normality. Data which followed a Gaussian distribution were compared using *t* test, chi‐square test, and ANOVA, whereas non‐parametric data were compared by Mann–Whitney U test, Friedman test, and Kruskal–Wallis H test with *p* < .05 considered as statistically significant. The core salivary microbiota was defined as bacterial genera and species present with a mean relative abundance >1% across all samples. The salivary microbiota were characterized and compared by means of predominant genera and species, relative abundance, α‐diversity (Shannon index), principal component analysis, and linear discriminant effect size analysis (Segata et al., 2011). Data on relative abundance were corrected for multiple dependent associations using Benjamini–Hochberg correction (Hochberg & Benjamini, 1990). All statistics were computed with MeV (Saeed et al., 2006) and GraphPad Prism (GraphPad).

## 3 RESULTS

### 3.1 Background and clinical data

Background data of the study population are detailed in Table 1. A comparable age and gender distribution was observed between the three groups. A significantly higher number of current smokers were identified in the periodontitis group (*p* < .05), whereas a significantly higher proportion of patients with psoriasis reported to attend regular dental care, as compared to periodontitis patients and healthy controls (*p* < .05). A limited proportion of the study population reported concurrent cardiovascular disease or diabetes. Patients with psoriasis were in good periodontal condition as expressed by number of teeth (mean: 26, range: 21–28), PPD (mean: 3.0, range: 2.4–3.4), and CAL (mean: 2.8, range: 2.0–3.9), despite having relatively high percentages of BOP (mean: 63, range: 26–99) and plaque (mean: 82, range: 37–100).

**Table 1:** Background data of the study groups

	Psoriasis (*n* = 27)	Periodontitis (*n* = 58)	Orally healthy controls (*n* = 52)
Gender male/female	16/11	33/25	27/25
Age (mean, range) years	55.3 (38–74)	53.4 (38–76)	54.8 (40–80)
Smoking status (Non‐/former/current smoker)	15/10/2*	20/15/23	30/16/6*
Heart disease (Y/N)	0/27	5/53	8/44
Diabetes (Y/N)	2/25	4/54	4/48
Regular dental care (Y/N)	23/4*	17/41	28/24

* *p* < .05.

### 3.2 Sequence metadata

A total number of 137 samples passed quality control with a mean (range) of 33,591 (8,278–105,689) sequences per samples. In general, 99.8 (96.2–100.0)% of the generated sequences could be identified at genus level, whereas 98.0% (93.7–99.8) of the sequences could be identified at species level. In total, 477 different taxa were identified. A comparable percentage of genus‐level and species‐level identifications were observed in all groups studied.

### 3.3 Core salivary microbiota in psoriasis, periodontitis, and oral health

**Figure 1:**
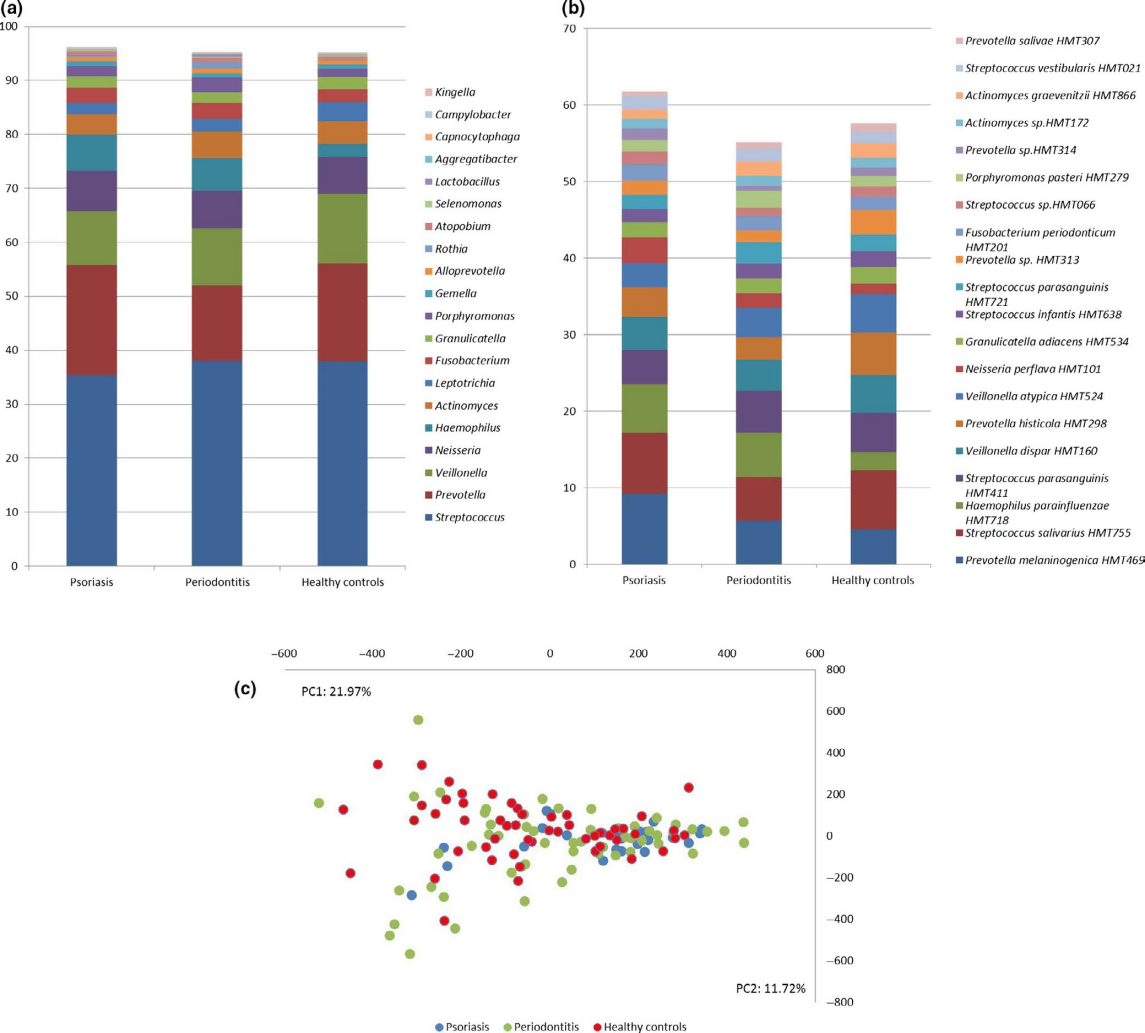
Core salivary microbiota. (a) Relative abundance of predominant bacterial genera expressed as % of total sequences. (b) Relative abundance of predominant bacterial species expressed as % of total sequences. (c) Principal component analysis with x (PC1) and y‐axis (PC2) as the two most decisive components collectively accounting for 33.7% of the variation. Sample denotation: psoriasis: blue, periodontitis: green, and oral health: red

The composition of the predominant bacterial genera and species identified is presented in Figure 1a‐b. The four most predominant bacterial genera identified in all three study groups, which constituted approximately 70% of the salivary microbiota, were *Streptococcus, Prevotella*, *Veillonella,* and *Neisseria*. At species level, the most predominant bacterial species was *Prevotella melalogenica* followed by *Streptococcus salivarius.* In general, the most predominant bacterial species identified were *Streptococcus*, *Prevotella,* and *Veillonella*. No significant differences were observed in α‐diversity and relative abundance of predominant genera or predominant species in the three groups. Furthermore, principal component analysis revealed a completely random distribution of saliva samples from patients with psoriasis, patients with periodontitis, and healthy controls (Figure 1c).

### 3.4 Relative abundance and linear discriminant effect size analysis of salivary microbiota

**Figure 2:**
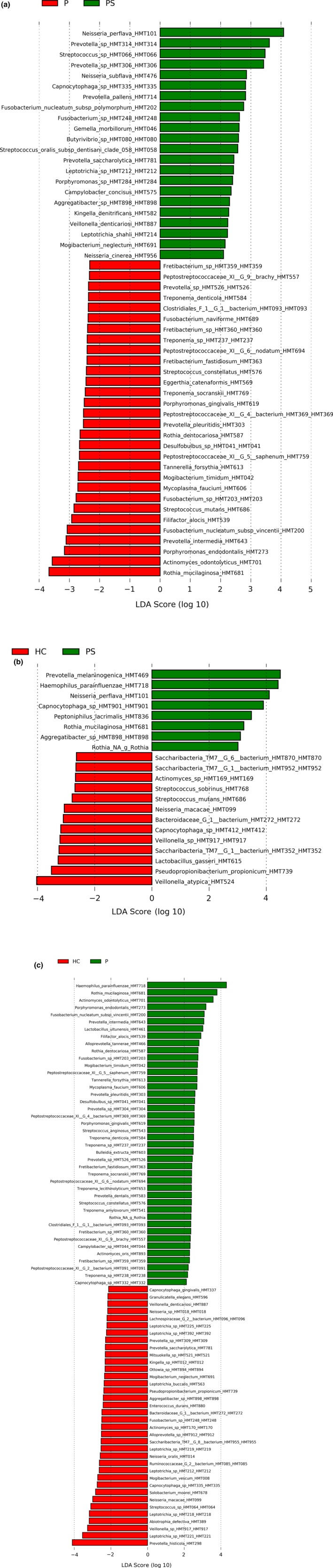
Linear discriminant effect size analysis. Linear discriminant analysis score expressed as (log 10) of significant bacterial taxa. (a) Psoriasis (PS) versus periodontitis (P). (b) Psoriasis (PS) versus healthy controls (HC). (c) Periodontitis (P) versus healthy controls (HC)

A total of 18 bacterial species were identified with a significantly different relative abundance in saliva samples from patients with psoriasis, patients with periodontitis, and oral healthy controls (Table 2, adjusted *p* < .001). A significantly higher relative abundance of specific proposed periodontal pathogens, such as *Porphyromonas gingivalis*, *Treponema denticola*, *Tannerella forsythia*, *Prevotella intermedia,* and *Filifactor alocis,* was identified in saliva samples from patients with periodontitis. Pairwise comparisons using linear discriminant effect size analysis showed that 52 (22 psoriasis‐associated and 30 periodontitis‐associated; Figure 2a) taxa differentiated patients with psoriasis from patients with periodontitis, 21 (8 psoriasis‐associated and 13 orally healthy control‐associated; Figure 2b) taxa differentiated patients with psoriasis from orally healthy controls, and 78 (42 periodontitis‐associated and 36 orally healthy control‐associated; Figure 2c) taxa differentiated patients with periodontitis from orally healthy controls, respectively.

**Table 2:** Bacterial taxa with significantly different relative abundance

	Psoriasis		Periodontitis		Orally healthy controls		*p*‐value
	RA	Presence	RA	Presence	RA	Presence	
Rothia mucilaginosa HMT681	0.330686	96.15385	1.064432422	100	0.051519542	88.46154	1.00E‐11
Mycoplasma faucium HMT606	0.002838	3.846154	0.075078827	72.41379	0.004729376	19.23077	3.93E‐10
Tannerella forsythia HMT613	0.011631	38.46154	0.07827513	82.75862	0.007009139	38.46154	2.74E‐08
Peptostreptococcaceae [XI][G‐6] nodatum HMT694	0.000757	3.846154	0.021276436	55.17241	0.00195116	7.692308	6.52E‐08
Porphyromonas gingivalis HMT619	0.000000	0	0.049856888	50	0.000804377	5.769231	1.22E‐07
Filifactor alocis HMT539	0.012395	15.38462	0.135676421	63.7931	0.015349738	21.15385	2.65E‐07
Rothia dentocariosa HMT587	0.022593	23.07692	0.091595477	65.51724	0.003578482	17.30769	7.87E‐07
Bacteroidaceae [G‐1] HMT272	0.005529	15.38462	0.001228553	6.896552	0.015004592	50	7.49E‐06
Treponema sp.HMT237	0.010219	15.38462	0.045178271	50	0.002571879	11.53846	8.10E‐06
Mogibacterium timidum HMT042	0.030495	46.15385	0.106152765	82.75862	0.026568563	53.84615	1.34E‐05
Fusobacterium nucleatum HMT200	0.066781	42.30769	0.257576702	77.58621	0.07180377	48.07692	3.15E‐05
Treponema denticola HMT584	0.009736	23.07692	0.040443669	48.27586	0.001278036	9.615385	4.14E‐05
Fretibacterium fastidiosum HMT363	0.005070	19.23077	0.032599761	53.44828	0.003339819	17.30769	4.14E‐05
Prevotella intermedia HMT643	0.034083	30.76923	0.244041221	62.06897	0.031503929	23.07692	5.05E‐05
Peptostreptococcaceae [XI][G‐5] saphenum HMT759	0.011885	11.53846	0.076931378	53.44828	0.007156812	23.07692	5.14E‐05
Porphyromonas endodontalis HMT273	0.090996	61.53846	0.319207693	87.93103	0.084759485	69.23077	6.89E‐05
Fretibacterium sp.HMT360	0.004001	19.23077	0.024301347	51.72414	0.002661645	15.38462	7.59E‐05
Peptostreptococcaceae [XI][G‐4] HMT369	0.001159	11.53846	0.008681835	44.82759	0.001356973	9.615385	1.60E‐04

Abbreviation: RA, relative abundance.

### 3.5 Salivary NGAL and transferrin levels

All samples were recorded with NGAL levels above detection limit, whereas transferrin levels were below detection limit in 30 samples.

A significantly lower mean salivary level of NGAL (996 [std. error 320] ng/ml) was recorded in patients with psoriasis, as compared to patients with periodontitis (2,072 [295] ng/ml) and orally healthy controls (2,551 [345] ng/ml, *p* < .0001).

Salivary transferrin levels were also significantly lower in patients with psoriasis (4.37 [0.92] ng/ml), as compared to patients with periodontitis (7.25 [0.88] ng/ml) and orally healthy controls (10.02 [0.94] ng/ ml, *p* < .0001).

## 4 DISCUSSION

The purpose of the present study was to characterize the composition of the salivary microbiota and measure salivary levels of NGAL and transferrin in patients with psoriasis, and compare these data to the characteristics in patients with periodontitis and orally healthy controls.

We found that psoriasis was associated with specific characteristics of the salivary microbiota, which were different from patients with periodontitis and orally healthy controls (Figure 2a‐b, Table 2). Furthermore, patients with psoriasis had significantly lower salivary NGAL and transferrin levels than patients with periodontitis and orally healthy controls. These data suggest that psoriasis impacts oral homeostasis and the significance of reduced levels salivary levels of NGAL and transferrin in psoriasis clearly also merits further study.

In the present study, 29% of the patients with psoriasis were excluded as they had treatment‐requiring periodontitis. This finding is in line with increased risk of periodontitis found in other studies (Skudutyte‐Rysstad et al., 2014; Woeste et al., 2019). In a recently published paper, the prevalence of periodontitis in a Danish cohort ranged from 9.2% to 31.0% depending on the periodontal classification used (Kongstad, Enevold, Christensen, Fiehn, & Holmstrup, 2017). Thus, data from the present study support a psoriasis‐associated risk of periodontitis. Notably, all patients with psoriasis, who were excluded based on the presence of treatment‐requiring periodontitis, reported to attend regular dental care, as compared to 54% of orally healthy controls, indicating that patients with psoriasis might be prone to periodontitis despite having regular dental care.

The core salivary microbiota identified in patients with psoriasis could not be differentiated from patients with periodontitis and orally healthy controls (Figure 1a‐c). However, 18 bacterial species were identified with a significantly different relative abundance in the three groups studies (Table 2). Notably, the majority of these bacterial species, including the proposed periopathogen *P. gingivalis,* were identified with higher relative abundance in patients with periodontitis. Salivary levels of *P. gingivalis* have been reported to associate with periodontitis (Damgaard et al., 2019), and previous studies have shown a positive correlation of salivary and subgingival levels of *P. gingivalis* (Belstrøm et al., 2018; Nickles, Scharf, Röllke, Dannewitz, & Eickholz, 2017) suggesting that the salivary microbiota reflects local bacterial alterations associated with periodontitis.

Using linear discriminant effect size analysis, we identified unique characteristics of the salivary microbiota in psoriasis, as 52 (22 psoriasis‐associated and 30 periodontitis‐associated; Figure 2a) and 21 (8 psoriasis‐associated and 13 health‐associated; Figure 2b) taxa differed in psoriasis as compared to periodontitis and orally healthy controls, respectively. To the best of our knowledge, this is the first study to characterize the salivary microbiota in patients with psoriasis. On the other hand, several studies have demonstrated an impact of diabetes on the composition of the oral microbiota (Wang et al., 2019; Xiao et al., 2017), and two recent studies reported that periodontal bone loss in patients with rheumatoid arthritis was associated with alterations of the oral microbiota (Corrêa et al., 2019, 2016). In addition, dysbiosis of the salivary microbiota has been linked with inflammatory bowel disease (Xun, Zhang, Xu, Chen, & Chen, 2018). Thus, accumulated evidence suggests that systemic diseases, as exemplified in the current study by psoriasis, might impact the composition of the salivary microbiota.

We found significantly lower salivary levels of NGAL and transferrin in patients with psoriasis, as compared to patients with periodontitis and orally healthy controls, respectively. To the best of our knowledge, salivary levels of these inflammation‐related proteins have not previously been reported in patients with psoriasis. However, a study from 2015 showed higher salivary levels of pro‐inflammatory cytokines, including tumor necrosis factor‐α, IL‐1β, transforming growth factor‐1β, and monocyte chemoattractant protein‐1 in patients with psoriasis as compared to orally healthy controls (Ganzetti et al., 2015). Therefore, psoriasis seems to associate with increased levels of inflammatory markers in saliva and it is remarkable that in our study patients with psoriasis had significantly lower levels of salivary NGAL as compared to patients with periodontitis and orally healthy controls, respectively. NGAL is an inflammation‐related protein, and increased levels of NGAL in the circulation and urine have been intensively investigated as a marker of acute kidney injury (Hjortrup, Haase, Treschow, Møller, & Perner, 2015). Also, in periodontitis a positive correlation has been found between urinary levels of NGAL and the severity of periodontitis (Nakajima et al., 2019). Notably, in a study from 1996, increased expression of NGAL was identified in gingival tissue and saliva from patients with periodontitis (Westerlund et al., 1996), and a recent report showed that experimental gingivitis caused an increase in salivary levels of NGAL (Morelli et al., 2014). Furthermore, higher NGAL levels in local samples, that is, gingival crevicular fluid (GCF), were reported in patients with periodontitis (Pradeep, Nagpal, Karvekar, & Patnaik, 2016). The present salivary NGAL data are therefore intriguing and warrant further study, but it is remarkable that conflicting data also exist for other salivary inflammatory markers in periodontitis, for example, with increased (Lee et al., 2018) or similar (Moura et al., 2017) salivary levels of IL‐1β compared to healthy controls. While the main contributor to salivary proteins is the circulation, with proteins shed from local oral surfaces playing a lesser role (Lynge Pedersen & Belstrom, 2019), disease‐specific mechanisms are likely to be involved, for example, with periodontitis‐dependent increased expression of inflammatory mediators in the inflamed oral tissue potentially being countered by increased local tissue binding or other interactions that contribute to unpredictability of salivary levels of these molecules. It would therefore be recommendable to include blood and GCF samples in future studies on salivary levels of inflammation‐related proteins.

Also, salivary transferrin levels were lower in patients with psoriasis compared to patients with periodontitis and orally healthy controls, respectively. Transferrin is a “negative” inflammation‐related protein, and inflammation is associated with a decrease in circulating transferrin levels. Accordingly, several studies have reported that periodontal treatment is associated with an increase in blood transferrin levels, albeit that to our knowledge, there is no evidence available on salivary transferrin levels (Fang et al., 2015; Shirmohamadi et al., 2016).

Some limitations apply to the present study, primarily the relatively low number of included patients with psoriasis. However, as significant differences in the salivary microbiota and salivary levels of NGAL and transferrin, respectively, were observed, the number of participants may have been sufficient. Furthermore, the severity of psoriasis and current treatment regimen was not recorded but we recently found that similar patients with psoriasis from the recruiting hospital had a psoriasis area and severity index (PASI) score of approximately 10 suggestive of severe disease (Ahlehoff et al., 2011). Moreover, as blood samples and local samples from the oral cavity, for example, GCF, were not collected, it was not possible to compare salivary, local, and circulating levels of NGAL and transferrin. In addition, the higher amount of current smokers in the periodontitis group may have influenced data on the salivary microbiota, as smoking status has been reported to influence the composition of the subgingival microbiota (Mason et al., 2015). However, previous studies have shown that the impact of smoking on the salivary microbiota is probably not as pronounced (Belstrøm, Fiehn, et al., 2014; Belstrøm, Holmstrup, et al., 2014). Finally, the prevalence of cardiovascular disease and diabetes in patients with psoriasis was in the present study somewhat lower than expected, which might be explained by the fact that cardiovascular disease and diabetes was self‐reported and therefore not based on data from a medical examination.

In conclusion, data from the present study suggest that psoriasis is associated with characteristics of the salivary microbiota and salivary levels of inflammation‐related proteins, which is different from that of patients with periodontitis and orally healthy controls. More studies are needed to shed further light on the mechanisms underlying the association between psoriasis and periodontitis.

## CONFLICT OF INTEREST

The authors have stated explicitly that there are no conflicts of interest in connection with this article. This study was supported financially by the Independent Research Council Denmark, the Danish Foundation for Mutual Efforts in Dental Care, and The Danish Dental Association. Daniel Belstrøm and Josefine Maria Eiberg contributed equally to this manuscript. PRH is supported by unrestricted grant from the LEO Foundation, a Borregaard Clinical Scientist Fellowship from the Novo Nordisk Foundation, and a clinical academic group grant from the Greater Copenhagen Health Science Partners.

## AUTHOR CONTRIBUTION

Daniel Belstrøm (DB), Peter Riis Hansen (PRH), and Claus Antonio Juel Jensen (CAJJ) designed the study. Josefine Maria Eiberg (JME) and Lone Skov recruited the patients. JME performed the clinical examinations and collected the samples. Christian Enevold, Maria Grande, and CAJJ performed the molecular analysis. DB did the statistical analysis. DB and PRH wrote the first draft of the manuscript, which was critically revised by all authors. All authors approved the final version of the paper.

## Data Availability

Unrestricted access to all data including raw sequences will be granted upon request to the corresponding author (dbel@sund.ku.dk).
